# The impact of acrylamide regulations on the competitive structure of the grain market^☆^

**DOI:** 10.1016/j.fochx.2025.103286

**Published:** 2025-11-13

**Authors:** Veronica Nowakowski

**Affiliations:** Goldman School of Public Policy, University of California, Berkeley, 2607 Hearst Avenue, Berkeley, CA 94720, USA

## Abstract

This paper examines the European grain market in the context of emerging food safety regulations targeting free asparagine accumulation in wheat and its role in acrylamide formation during thermal processing. The wheat market is often characterized as near-competitive, with standardized commodities, transparent price discovery, and a dispersed producer base. However, stricter acrylamide standards have the potential to alter this structure. While these regulations aim to reduce consumer exposure to a recognized process contaminant, they may unintentionally reinforce market concentration by privileging firms with the capital and testing infrastructure required for compliance. Small and mid-sized millers and manufacturers face comparatively higher costs in sourcing low-asparagine wheat and adopting monitoring systems, raising barriers to entry. By analyzing market organization, contract arrangements, and the limited scope for product differentiation, this paper evaluates how regulatory asymmetries may constrain competition and offers policy recommendations to support both effective food safety governance and equitable market access.

## Highlights

• Acrylamide rules may accelerate consolidation in the EU grain market
• Small firms face sourcing, testing, and supply chain access challenges
• Large firms control supply chains through contracts and custom grain sourcing
• Acrylamide rules risk disrupting grain supply chains for small manufacturers
• Policy support is needed to protect food supply chain diversity and equity

## 1 Introduction

### 1.1 Background on the grain market

Grains are a fundamental commodity in the global food supply chain, playing a central role in both human consumption and animal feed. Products like wheat, corn, barley, and rye are traded extensively across countries and regions, forming the backbone of many agricultural economies. Due to their essential nature and widespread production, grain markets have developed into highly organized systems where global supply and demand drive pricing and trade policies. These markets are especially crucial in regions like Europe, where a significant portion of agricultural land is dedicated to grain cultivation, and where trade blocs such as the European Union influence market dynamics through regulations and subsidies (European Commission, 2021, OECD& FAO; & FAO, 2023, Tomek & Kaiser, 2014; European Commission/Eur-Lex, 2018).

Grain markets have historically aligned closely with the economic model of perfect competition. In early agricultural societies, local farmers produced grain for their own communities. But as transportation improved and trade networks expanded, grain became one of the first commodities to be traded on a global scale. By the 19th and 20th centuries, especially with the establishment of futures markets in Chicago and Paris, grain prices became increasingly influenced by transparent, competitive bidding systems (Carter, Rausser, & Smith, 2017). These developments laid the foundation for a grain market characterized by many producers and buyers, homogeneous products, and prices determined largely by supply and demand (Wright, 2011).

The grain market closely reflects near-perfect competition. Entry is relatively accessible, and grains are standardized commodities (e.g., hard red winter wheat, feed barley), which enables direct price comparisons (Tomek & Kaiser, 2014). Prices are published transparently through futures exchanges and commodity boards, reducing monopolistic behavior (Ghoshray, 2013).

In Europe, the grain market plays a central role in both domestic agriculture and international trade. The European Union is one of the world's largest grain producers, with France, Germany, and Romania leading production (OECD& FAO; & FAO, 2023). The Common Agricultural Policy (CAP) has historically shaped the grain market by offering subsidies and support programs to farmers, thereby influencing production levels and stabilizing incomes (Matthews, 2016). Additionally, the EU maintains strict quality standards and grading systems that support product standardization across member states. Price transparency is facilitated through organizations like the European Commission's Directorate-General for Agriculture and Rural Development, which publishes regular market updates (European Commission, 2023). While the CAP has created some distortions compared to a textbook perfectly competitive market, the European grain sector still retains many characteristics of a highly competitive environment.

Acrylamide formation in food is closely linked to the presence of free asparagine, a naturally occurring amino acid found in grains like wheat. When foods containing both free asparagine and reducing sugars are subjected to high temperatures, such as during baking, roasting, frying, or toasting, a chemical reaction known as the Maillard reaction can occur, resulting in the formation of acrylamide. Acrylamide has been classified by health agencies as a probable human carcinogen (IARC Monographs, Vol. 60, 1994), prompting regulatory efforts to limit its presence in processed foods (Gökmen & Şenyuva, 2022; Mottram, Wedzicha, & Dodson, 2002; Xu, Oruna-Concha, & Elmore, 2020). Because free asparagine is the key precursor to acrylamide formation in cereals, many food safety interventions target the reduction of free asparagine levels in raw grain as a way to control acrylamide content in the final food product (Halford, Curtis, Muttucumaru, Postles, & Mottram, 2007; Deribew & Woldegiorgis, 2021).

The implementation of strict acrylamide regulations may alter market competition by disproportionately burdening smaller and mid-sized manufacturers while favoring large corporations. While intended to improve public health and food safety, a critically important goal, such regulations could unintentionally shift the grain-based product market, particularly in the European Union, toward further consolidation, reducing competition and limiting the diversity of producers able to comply with evolving food safety standards. This paper does not oppose regulation itself, but argues that it should be paired with equitable implementation strategies, notably to support smaller and mid-sized firms that play a vital role in food system diversity and innovation.

## 2 Characteristics of the competitive grain market

### 2.1 Perfect competition in the wheat market

The wheat market exemplifies the economic concept of perfect competition, a market structure characterized by a large number of buyers and sellers who individually lack the power to influence prices. Because wheat is cultivated in many countries and by producers of varying scales—from smallholder farms in Eastern Europe to industrial agribusinesses in France or Germany—no single actor can unilaterally manipulate the market. Instead, wheat prices are largely determined by aggregate supply and demand trends across local, regional, and global levels. This decentralized pricing mechanism allows for a high degree of transparency and minimizes the potential for monopolistic behavior. As a result, wheat producers must remain price-takers, meaning they accept prevailing market prices rather than set them themselves (Tomek & Kaiser, 2014; Carter et al., 2017). In this environment, competition is not based on branding or loyalty but on efficiency and the ability to produce a standardized product that meets established quality criteria.

This system is reinforced by the relatively homogeneous nature of wheat as a product. Unlike fruits or vegetables, which may vary in taste, appearance, and branding, wheat is graded based on technical and measurable qualities such as protein content, test weight, and moisture level. These grades are established by national and international organizations—like the USDA and the European Commission—ensuring that wheat of the same grade is virtually interchangeable across producers and regions (European Commission, 2023; FAO, 2022). This product uniformity enables a highly competitive market because buyers can easily compare offers from different sellers and base purchasing decisions purely on price and logistical factors like transportation or availability. Consequently, innovation or branding plays a limited role in raw wheat sales, reinforcing the market's alignment with the perfect competition model.

### 2.2 Traditional market dynamics

The structure and efficiency of the wheat market are further shaped by the role of futures contracts and spot markets. Futures contracts—standardized agreements to buy or sell a specific quantity of wheat at a predetermined price on a future date—are traded on major exchanges such as the Chicago Board of Trade and Euronext. These financial instruments provide producers and buyers with a powerful tool for managing risk, particularly price volatility that can result from weather events, geopolitical disruptions, or changes in input costs like fertilizer or fuel (Wilson et al., 2020) Futures markets also contribute to price discovery, enabling transparent and real-time tracking of supply-and-demand expectations well in advance of physical delivery. This forecasting function is especially important in the grain sector, where planting and harvesting decisions are made months before actual sales occur (Wright, 2011).

In addition to futures markets, a vast network of intermediaries facilitates the day-to-day operations of the wheat market. Grain elevators, cooperatives, and local aggregators collect and store wheat, perform quality grading, and consolidate shipments for bulk transport. These intermediaries serve as a bridge between producers and larger-scale buyers, such as flour mills, exporters, and food processing companies. For small and mid-sized farmers who may lack the infrastructure or market access to negotiate with major buyers directly, these intermediaries are essential. They not only facilitate physical transactions but also help ensure that wheat meets the quality standards necessary for entering domestic and international markets. In many regions of Europe, farmer cooperatives also play a significant role in strengthening bargaining power and increasing market access for rural producers (Tomek & Kaiser, 2014).

### 2.3 Lack of product differentiation in raw grain

Despite its economic significance and global reach, the wheat market remains marked by a limited degree of product differentiation at the raw commodity level. Buyers primarily focus on a narrow set of quantifiable and verifiable characteristics—namely protein content, test weight, moisture level, and Falling Number (Hagberg Falling Number)—because these traits directly influence the milling and baking quality of the grain. These metrics have long been integrated into national and international grading standards, and they determine whether wheat is classified for food use, feed use, or other industrial applications. As such, producers have limited opportunities to distinguish their product unless they can consistently meet the specifications of higher-grade wheat, which typically commands a price premium (Ghoshray, 2013).

While this system promotes consistency and efficiency, it also creates barriers to innovation, particularly for efforts to introduce new quality attributes that fall outside the traditional grading framework. One such emerging trait is the reduction of free asparagine content in wheat. Free asparagine, when exposed to high-temperature cooking such as baking or frying, reacts with sugars to form acrylamide—a probably carcinogenic compound. Therefore, reducing free asparagine at the grain level can significantly lower acrylamide formation in food products (Curtis et al., 2009). Despite its public health significance, the market currently does not reward this trait, as it is not yet incorporated into grading systems or recognized by price premiums. This disconnect makes it difficult for breeders and producers to justify the additional costs associated with developing or cultivating low-asparagine wheat varieties. Without regulatory mandates or clear market incentives, these innovations remain niche and under-adopted. As health and food safety regulations—such as those concerning acrylamide—evolve, the challenge will be to reconcile the need for public benefit with the market's strong preference for standardization and pricing simplicity (OECD& FAO; & FAO, 2023).

## 3 Regulatory changes and their impact on market participants

### 3.1 Compliance strategies for large corporations

As food safety regulations evolve, particularly concerning acrylamide formation in grain-based products, large agribusinesses and food manufacturers have begun to proactively adopt compliance strategies that reflect their greater access to capital, influence, and logistical infrastructure. One common strategy is direct contracting with farmers to secure a reliable supply of low-asparagine grain. These contracts often specify growing conditions, input use, and target quality attributes—including low free asparagine content, which reduces acrylamide formation—to ensure that the grain entering the supply chain can meet regulatory thresholds. This allows corporations to bypass uncertainties in the spot market and maintain consistency in raw material quality across their global operations (European Commission, 2023; Matthews, 2016).

In addition, large firms are investing heavily in research and development aimed at reducing acrylamide content at the source (Friedman, 2015). This includes funding for selective breeding and biotechnology projects that target genetic markers associated with lower free asparagine production in wheat. These initiatives are often carried out in collaboration with academic institutions or seed companies and represent a long-term strategy for ensuring regulatory compliance while maintaining processing efficiency and end-product quality (OECD& FAO; & FAO, 2023; Pingali, 2012). Beyond breeding, large corporations also exert influence over agronomic practices by prescribing specific fertilizer regimes—such as maintaining moderate nitrogen and sufficient sulfur applications—as well as irrigation methods and crop rotations known to reduce free asparagine accumulation (Curtis et al., 2009; Corol et al., 2016; Jalli et al., 2021; Darguza and Gaile, 2020). These upstream interventions give them granular control over compliance risks and support branding efforts that promote food safety and quality assurance.

### 3.2 Challenges for Small and mid-sized manufacturers

In contrast, small and mid-sized manufacturers face a far more constrained set of options when attempting to comply with emerging acrylamide regulations. Many of these businesses rely on the spot market for their grain sourcing, purchasing raw wheat based on availability and price rather than long-term quality guarantees. This makes it difficult for them to consistently secure low-asparagine grain, especially in the absence of a widespread grading system that includes this attribute (Ghoshray, 2013). Without the ability to contract directly with growers or influence farming practices, smaller manufacturers remain largely reactive to supply-side changes, exposing them to greater risk of non-compliance and potential regulatory penalties.

Moreover, these manufacturers often lack the financial flexibility to absorb the additional testing costs and logistical burdens required to ensure compliance. Verifying free asparagine content adds an extra layer of lab analysis, supply chain tracking, and documentation that can be prohibitively expensive, particularly for firms operating with narrow margins. Unlike larger competitors, smaller producers may not be able to invest in on-site testing infrastructure or develop partnerships with laboratories, increasing their reliance on third-party certifications and trust in suppliers. If low-asparagine grain begins to command a premium in the marketplace—as is likely under stricter regulatory enforcement—small and mid-sized firms may struggle to remain cost-competitive without passing those costs onto consumers, risking a loss of market share (FAO, 2022).

Ultimately, this regulatory asymmetry raises concerns about equity in market participation. While larger firms are well-positioned to navigate or even shape regulatory compliance pathways, smaller actors may be pushed out or forced to reduce product offerings. Unless policy interventions explicitly address these gaps—such as subsidies for testing, transitional support for grain traceability, or regional certification schemes—there is a real risk that public health regulations intended to improve food safety will inadvertently contribute to market consolidation and reduce the diversity of food producers in the grain sector.

## 4 Potential market outcomes and unintended consequences

### 4.1 Market consolidation risks

While motivated by public health concerns, the implementation of strict acrylamide regulations in grain-based food products has the potential to significantly reshape the competitive landscape of the food manufacturing industry, primarily by accelerating market consolidation. Large corporations, with the financial capacity to invest in long-term supply contracts, research and development, and advanced testing infrastructure, will likely secure a growing share of the market for low-asparagine grain. These firms could use their market power to establish exclusive agreements with farmers or seed companies to ensure access to proprietary low-asparagine wheat varieties. By vertically integrating their operations—controlling everything from seed genetics to final product packaging—these corporations could reduce their exposure to regulatory uncertainty while simultaneously locking out smaller competitors who rely on open-market access to grain (OECD& FAO; & FAO, 2023; Matthews, 2016).

This trend toward consolidation creates substantial barriers to entry for small and mid-sized firms, which often lack the resources to navigate the increasing complexity of the regulatory environment. As the cost of compliance grows—through testing, traceability, and quality assurance—so too does the threshold for participation in the market. Independent bakeries, niche product lines, and regional food producers may be forced to either exit the industry or drastically limit their operations. These shifts are not just economic in nature; they also affect the social fabric of food systems by threatening local food traditions, community-based enterprises, and decentralized agricultural value chains. Over time, the consolidation of power among a few dominant players may result in decreased innovation, lower price competitiveness, and increased dependency on corporate-controlled food sources, with long-term implications for food sovereignty and market resilience (Ghoshray, 2013).

### 4.2 Impact on consumer prices and product diversity

The rising costs associated with regulatory compliance do not exist in a vacuum—they are ultimately passed down the supply chain and absorbed, at least in part, by consumers. When firms incur higher expenses to source low-asparagine grain, conduct lab testing, or reconfigure production processes, these costs are often embedded into the final retail price of food products. As a result, consumers may see increases in the price of everyday staples such as bread, pasta, breakfast cereals, and snack items. This effect is likely to be more pronounced in countries with higher levels of grain consumption or among households with constrained budgets, amplifying food insecurity and making nutrient-dense products less affordable for vulnerable populations (FAO, 2022).

Beyond price increases, regulatory burdens may also contribute to a noticeable reduction in product diversity on store shelves. Smaller manufacturers, who often drive culinary innovation and serve niche markets—such as gluten-free, organic, artisanal, or culturally specific food items—may find themselves unable to compete under the new regulatory regime. Their inability to absorb compliance costs or guarantee grain quality could force them to discontinue certain product lines or exit the market entirely. This contraction of market diversity diminishes consumer choice and erodes the richness of regional food cultures. It also stifles experimentation, as smaller firms typically serve as incubators for new product ideas and sustainable food practices that are later scaled by larger players. In this way, the consequences of acrylamide regulation may extend well beyond safety outcomes, reshaping consumer access, market aesthetics, and the very character of the modern food landscape.

### 4.3 Economic and trade considerations

At the international level, the uneven rollout of acrylamide regulations introduces a new layer of complexity to agricultural trade. Yet this challenge is not unique to acrylamide. Divergence in regulatory standards has long shaped the movement of food and agricultural products, from pesticide residue limits and maximum contaminant thresholds to country-specific labeling and certification schemes. These mismatches already function as non-tariff barriers that require costly adjustments from exporters, and acrylamide restrictions risk reinforcing and expanding those dynamics.

Nations that proactively implement stringent limits—such as EU member states—may restrict access for exporters in less-regulated regions, who must invest in new production methods, testing infrastructure, or documentation systems to remain competitive. Developing nations are particularly vulnerable, as many lack the laboratory capacity or agricultural extension systems to monitor compliance across supply chains. The result is a heightened risk of trade distortions that could undermine global agreements and spark disputes in forums such as the World Trade Organization (European Commission, 2023).

Moreover, inconsistent regulations across jurisdictions may lead to a phenomenon known as regulatory arbitrage, where firms strategically source grain from countries with looser standards to reduce costs. While this approach may allow businesses to remain competitive, it can also undermine the public health goals of the regulation and create uneven playing fields for producers operating under stricter guidelines. If grain labeled as compliant in one country is reprocessed or relabeled in another, the traceability of free asparagine content becomes murky, potentially reducing consumer trust and weakening enforcement mechanisms. Over time, this could fragment the global grain market into parallel systems—one for highly regulated regions and one for the rest—leading to inefficiencies, loss of transparency, and decreased cooperation on global food safety initiatives (OECD& FAO; & FAO, 2023). Without international harmonization efforts, such as shared testing protocols or mutual recognition agreements, regulatory inconsistency could hinder trade, suppress innovation, and amplify inequities between nations.

Without stronger international harmonization—whether through shared testing protocols, mutual recognition agreements, or enhanced transparency mechanisms—these divergences risk not only hindering trade but also suppressing innovation and widening inequities between nations. Acrylamide thus serves as both a pressing case and a window into the broader, recurring challenges of aligning food safety regulation in a globalized economy. While divergent regulatory standards pose significant challenges at the international level, an additional layer of complexity emerges in how these rules are implemented domestically, particularly regarding who bears the responsibility for compliance and how quickly firms are expected to adapt.

### 4.4 Implementation challenges

A further issue relates to how regulatory responsibility is distributed along the food supply chain. Current frameworks primarily place the onus for compliance on food manufacturers, who must invest in testing, reformulation, and labeling to meet acrylamide limits. This consumer-facing accountability has the advantage of immediacy but may reduce incentives for upstream actors, such as plant breeders, to accelerate the development of low-asparagine grain varieties that offer a longer-term and more systemic solution.

Another challenge is the short lead time between the publication of regulatory limits and their enforcement. Firms often have only a narrow window to renegotiate contracts, adapt processing methods, or secure access to accredited laboratories. While large corporations may have the resources to absorb these shocks, small and mid-sized enterprises face disproportionate burdens, as they typically lack the logistical flexibility or capital reserves to respond quickly. Without adequate transition periods or targeted support mechanisms, accelerated implementation risks narrowing the diversity of firms able to compete, inadvertently consolidating market power among the largest actors. These implementation challenges underscore the need for regulatory frameworks that not only set ambitious public health targets but also provide equitable pathways for all stakeholders to achieve compliance.

## 5 Policy recommendations and mitigation strategies

### 5.1 Phased implementation of regulation

One of the most critical recommendations for avoiding disproportionate market disruption is the adoption of a phased approach to enforcement. Although acrylamide regulations are grounded in legitimate public health concerns—particularly their potential link to carcinogenic outcomes—abrupt implementation risks destabilizing entire supply chains. Policymakers should therefore design regulatory rollouts that gradually introduce stricter thresholds over a multi-year period. This tiered approach would allow breeders to continue developing low-asparagine wheat varieties, give farmers the necessary time to adjust agronomic practices, and allow manufacturers to adapt sourcing strategies, lab testing procedures, and quality control infrastructure. Gradual enforcement can mitigate panic-buying, reduce the likelihood of sudden price shocks, and prevent the bottlenecks that often accompany rushed regulatory mandates (Wright, 2011). Such an approach balances the urgency of public health protection with the practical realities of supply chain adaptation and market stability.

### 5.2 Support mechanisms for smaller manufacturers

Even with phased regulation, small and mid-sized manufacturers remain the most vulnerable to compliance pressures. These firms often lack access to the long-term supply contracts, integrated logistics, and capital reserves that allow larger corporations to adapt quickly. To prevent the erosion of diversity within the food manufacturing sector, targeted support mechanisms must be embedded into regulatory frameworks. One avenue is the provision of subsidies or direct grants that offset the substantial costs of testing and certifying both grain and finished products for free asparagine levels. Importantly, such support should not be limited to initial compliance costs but should also extend to ongoing operational expenses, including routine laboratory analyses, certification renewals, and compliance audits. For example, the European Commission could establish a dedicated fund under the Common Agricultural Policy (CAP) designed specifically to assist smaller-scale food producers. Such a mechanism would allow these producers to comply without abandoning product lines or laying off workers, thereby protecting employment and product diversity. Additionally, promoting crop rotation practices—such as alternating cereals with legumes, oilseed rape, or other break crops—can help lower free asparagine accumulation in wheat and other grains (Curtis et al., 2018; Postles et al., 2013).

Equally important is democratizing access to low-asparagine grain. Currently, large corporations maintain an advantage by securing low-asparagine grain through direct contracts with farmers and vertical integration strategies. In contrast, smaller firms, which rely heavily on more volatile spot markets, are disadvantaged in sourcing the raw materials necessary for compliance. To address this imbalance, governments and nonprofit organizations could establish regional exchanges or digital platforms that aggregate and list certified low-asparagine grain sources. These platforms should be complemented by logistical infrastructure such as regional storage facilities, small-batch delivery services, and accessible quality testing hubs. Partnerships with agricultural extension services and rural development agencies could provide additional technical training to ensure both producers and processors understand how to participate in compliant supply chains. Without these access points, even financial subsidies would fail to address the structural challenge of grain availability, further marginalizing small businesses and eroding local food economies.

### 5.3 Encouraging fair competition

While financial and logistical assistance is vital, fostering fair competition within the grain and food manufacturing sectors requires proactive structural reforms that ensure regulatory benefits are not captured solely by the most powerful firms. One way to achieve this is through upstream incentives for farmers who produce low-asparagine grain. However, these incentives must be designed with equity in mind. If price supports or subsidies are delivered only through contracts with major corporations, small and mid-sized manufacturers will continue to face exclusion from compliant supply. Instead, governments could provide performance-based payments or sustainability bonuses to farmers who grow low-asparagine varieties while agreeing to sell through open or cooperative marketplaces. These payments could be structured to reward diversification, including practices that benefit soil health and crop resilience—such as crop rotation techniques that also reduce free asparagine accumulation—making the incentives part of a broader sustainable agriculture strategy (OECD& FAO; & FAO, 2023).

Beyond producer-side incentives, government-backed cooperatives offer a pathway to counterbalance corporate dominance and empower smaller food producers. These cooperatives could take the form of purchasing alliances, testing and quality control hubs, or regional branding collectives that highlight compliant yet independently produced food products. For example, a national or EU-wide cooperative model could allow small bakeries and grain processors to pool their resources to access compliant wheat in bulk, share laboratory equipment, or jointly contract with farmers for low-asparagine grain varieties. These shared models of ownership and operation have long histories in agriculture, particularly across Europe, where they have proven effective in improving the bargaining power of small producers and stabilizing rural economies (European Commission, 2023). Embedding regulatory compliance support into these networks would not only protect diversity in the food industry but also reinforce economic justice and regional resilience.

Voluntary compliance programs offer another important tool in the policy toolkit, particularly as transitional strategies before full regulatory enforcement begins. These programs can take several forms, such as labeling initiatives, pilot certification systems, and public-private partnerships that recognize and reward early adopters. Through such programs, firms would be encouraged—not compelled—to meet the desired health and safety standards, often receiving marketing benefits, consumer trust, and perhaps small tax or subsidy advantages in return. Over time, successful voluntary programs can help create cultural and industry momentum that makes formal regulation more acceptable and less burdensome. Moreover, they provide regulators with valuable data on industry readiness, technical feasibility, and enforcement bottlenecks, enabling better-informed policy decisions later on. In the context of acrylamide, a voluntary “low-acrylamide certified” label could help consumers identify safer food options and allow smaller companies to gain competitive ground through innovation and commitment to health—even without the infrastructure of a multinational corporation.

However, designing such a certification system presents significant technical difficulties. Regulators must first define what qualifies as “low,” recognizing that acrylamide levels vary across food types and processing methods. A fixed maximum limit may be appropriate for some products but unrealistic for others, making the establishment of flexible yet consistent thresholds essential. A further complication lies in determining whether to regulate at the grain-input stage (e.g., acceptable free asparagine intervals) or in the finished product. Measurement methods also pose challenges: while high-performance liquid chromatography (HPLC) and gas chromatography–mass spectrometry (GC–MS) are accurate, they remain costly and technically demanding, particularly for smaller firms (Adimas et al., 2024, Corol et al., 2016, Lupăescu et al., 2025). Without harmonized, affordable testing protocols, certification risks becoming inaccessible or unreliable. Finally, regulators must decide how certification will be overseen—whether by government agencies, independent third parties, or industry groups—since each model carries different trade-offs in cost, credibility, and scalability. These complexities underline the importance of carefully designing voluntary programs to ensure that labels are both scientifically valid and trusted by consumers.

### 5.4 Balancing public health with market stability

Ultimately, the goal of acrylamide regulation must be to align public health protection with economic inclusivity. Striking this balance requires adaptive regulation that accounts for differences in firm size, market access, and supply chain integration. Phased implementation gives all actors time to adjust, targeted subsidies and logistical supports preserve diversity, and cooperative models counterbalance the dominance of large corporations. Voluntary compliance programs further ease the transition while building consumer awareness and industry momentum. Taken together, these strategies can transform acrylamide regulation from a potential driver of market consolidation into an opportunity for building a safer, fairer, and more resilient food economy.

## 6 Conclusion

Stricter acrylamide regulations are scientifically and ethically justified, yet they risk reshaping the grain industry in ways that privilege large corporations and disadvantage smaller producers. Firms with greater resources and vertically integrated supply chains can absorb compliance costs, while small and mid-sized manufacturers, often operating on thin margins, face barriers that could reduce their participation in the market. Without support, these dynamics may narrow product diversity, undermine cultural food traditions, and stifle innovation.

To prevent consolidation and ensure that regulation strengthens rather than fragments food systems, policymakers should design measures that strike a balance between safety and equity. Phased implementation, targeted subsidies, cooperative sourcing models, and voluntary compliance programs can ease adjustment and preserve competitiveness. Ultimately, acrylamide regulation should be viewed not only as a public health intervention but as an opportunity to build a food economy that is healthier, more inclusive, and resilient. Thoughtful design will determine whether these rules become a tool for exclusion or a precedent for equitable food policy.

## CRediT authorship contribution statement

**Veronica Nowakowski:** Writing – review & editing, Writing – original draft, Validation, Resources, Project administration, Methodology, Investigation, Formal analysis, Conceptualization.

## Declaration of competing interest

The authors declare the following financial interests/personal relationships which may be considered as potential competing interests: Given their role as Guest Editor, Dr. Christine Nowakowski had no involvement in the peer-review of this article and has no access to information regarding its peer-review. Dr. Christine Nowakowski is the mother of the author of this article. Due the this familial relationship and potential bias/conflict of interest in favor of the author, full responsibility for the editorial process for this article was delegated to another journal editor. If there are other authors, they declare that they have no known competing financial interests or personal relationships that could have appeared to influence the work reported in this paper.

## Data Availability

No data was used for the research described in the article.
